# Data set on Rapid Diagnostic Tests (RDTs) and microscopy for diagnosing plasmodium falciparum and plasmodium vivax

**DOI:** 10.1016/j.dib.2018.08.032

**Published:** 2018-08-15

**Authors:** Grace I. Olasehinde, Uche C. Oyeka, Margaret I. Oniha, Olabode A. Onile-ere, Olayemi O. Ayepola, Adesola A. Ajayi, Louis O. Egwari

**Affiliations:** Department of Biological Sciences, Covenant University, Ota, Ogun State, Nigeria

## Abstract

The World Heal Organization (WHO) has identified malaria diagnosis as being pivotal to eradicating the disease by 2030 as stipulated in the Sustainable Development Goals (SDG). The data presented here was obtained from outpatients of a hospital in the South Western Region of Nigeria from November 2016 to May 2017. The data contains malaria incidence amongst asymptomatic and symptomatic outpatients in the period under review. Malaria incidence was obtained using two diagnostic test kits, Bioline SD (HRP-2) and ACON (HRP-2/Aldolase) alongside Microscopy as gold standard. Specificity, Sensitivity and Kappa statistic of each test device is presented in the tables herewith. Data presented here could be used alongside other data sources to assess the state of malaria diagnostics.

**Specifications Table**

**Table:** 

Subject area	*Microbiology, Parasitology*
More specific subject area	*Malaria Diagnosis and Control*
Type of data	*Table*
How data was acquired	*Microscopy; Rapid Diagnostic Methods*
Data format	*Raw, Analyzed data in tables*
Experimental factors	*Ethical approval obtained from BIOSREC, Consent and accent sought*
Experimental features	*Blood samples obtained from febrile and non-febrile subjects visiting the selected health centers and tested for malaria using different methods*
Data source location	*Ado Odo/Ota, Ogun State, Nigeria*
Data accessibility	*Data is available in article*
Related research article	Dozie and Chukwuocha [4]*Comparative Evaluation of Malaria Rapid Diagnostic Test Kits Commercially Available in Parts of South Eastern Nigeria J Trop Dis 04(02)*[4]

**Value of the data**

• Data provided here could inform the quality and choice of Rapid Diagnostic Test (RDT) in malaria endemic regions.
• Data presented here, when compared with data from other regions, could be used to measure the efficacy of RDTs vis-à-vis the malaria control agenda.
• Data provided may inform the development of cheap and non-invasive diagnostic method for malaria.

## 1 Data

Table 1, Table 2, Table 3, Table 4 present data from diagnosis using the RDT kits as well as microscopy among asymptomatic participants. The tables contain the counts for total number of participants alongside the number that tested positive. Table 5, Table 6, Table 7, Table 8 present results obtained for the RDTs and microscopy among participants who were symptomatic i.e. had a fever ≥ 37.5 °C. Table 9, Table 10, Table 11, Table 12 contains a summary assessment of both RDT kits tested, using microscopy as baseline).

**Table 1:** Incidence of *P. falciparum* using Bioline SD kits among asymptomatic carriers.

Age group in years	No of samples collected			No of positive samples on kit (BIOLINE SD)		
	Male	Female	Total	Male	Female	Total
0–5	35	43	78	9	13	22
6–11	10	9	19	2	1	3
12–17	6	11	17	–	3	3
18–23	8	12	20	1	1	2
24–29	12	19	31	–	2	2
30	17	18	35	1	1	2
Total (%)	88 (44)	112 (66)	200 (100)	13 (6.5)	21 (10.5)	34 (17)

**Table 2:** Incidence of *P. falciparum* using ACON kits among asymptomatic carriers.

Age group in years	No of samples collected			No of positive samples in kit (ACON)		
	Male	Female	Total	Male	female	Total
0–5	35	43	78	6	8	14
6–11	10	9	19	3	3	6
12–17	6	11	17	–	4	4
18–23	8	12	20	2	2	4
24–29	12	19	31	1	4	5
30	17	18	35	–	3	3
Total (%)	88 (44)	112 (66)	200 (100)	12 (6)	24(12)	36(18)

**Table 3:** Incidence of *P. falciparum* using microscopic method among asymptomatic carriers.

	No of samples collected			No of positive samples		
	Male	Female	Total	Male	Female	Total
0–5	35	43	78	12	16	28
6–11	10	9	19	2	3	5
12–17	6	11	17	4	3	7
18–23	8	12	20	3	4	7
24–29	12	19	31	–	4	4
30	17	18	35	1	1	2
Total (%)	88 (44)	112 (66)	200 (100)	22 (11)	31 (15.5)	53 (26.5)

**Table 4:** Comparative incidence rates of malaria among asymptomatic subjects using ACON, Bioline SD kits and microscopy.

Sex	Number of sample collected (%)	Number of Positive samples (%)		
		Microscopy	Bioline SD	ACON
Male	88 (44)	22 (25)	13 (14.8)	12(13.6)
Female	112 (56)	31 (27.7)	21 (18.8)	24(21.4)
Total	200 (100)	53 (26.5)	34 (17)	36(18)

**Table 5:** Incidence of *P. falciparum* and *P. vivax* using ACON RDT kit among symptomatic subjects.

Age group in years	Number of samples collected			Number of positive samples					
				Male		Female		Total	
	Male	Female	Total	P.f	P.v	P.f	P.v	P.f	P.v
0–5	1	–	1	–	–	–	–	–	–
6–10	1	1	2	1	–	–	–	1	–
11–15	–	4	4	1	–	–	–	1	–
16–20	11	16	27	7	2	9	–	16	2
21–25	3	4	7	2	–	1	–	3	–
>26	9	10	19	–	–	2	–	2	–
Total	25 (41.7)	35 (58.3)	60 (100)	11 (47.8)	2(100)	12(52.2)	-(-)	23(100)	2(100)

**Table 6:** Incidence of *P. falciparum* using Bioline SD kits among symptomatic subjects.

Age group in years	Number of samples collected			Number of positive samples		
	Male	Female	Total	Male	Female	Total
0–5	1	–	1	–	–	–
6–10	1	1	2	1	–	1
11–15	–	4	4	1	1	2
16–20	11	16	27	7	9	16
21–25	3	4	7	2	1	3
>26	9	10	19	–	2	2
Total	25(41.7)	35(58.3)	60(100)	11 (45.8)	13(54.2)	24(100)

**Table 7:** Incidence of *P. falciparum* and *P. vivax* using microscopy among symptomatic subjects.

Age group in years	Number of samples collected			Number of Positive samples		
	Male	Female	Total	Male	Female	Total
0–5	1	–	1	1	–	1
6–10	1	1	2	1	–	1
11–15	–	4	4	–	–	–
16–20	11	16	27	10	9	19
21–25	3	4	7	–	–	–
>26	9	10	19	4	–	4
Total	25(41.7)	35(58.3)	60 (100)	16(64)	9 (36)	25 (100)

**Table 8:** Incidence of malaria using ACON kits, Bioline SD kits and microscopy among symptomatic subjects.

Sex	Number of samples collected (%)	Number of Positive Samples (%)			
		Microscopy (%)	RDTs		
			ACON		Bioline SD (HRP-2) (%)
			HRP-2 (%)	Pan-Aldolase (%)	
Male	25 (41.7)	16 (64)	11 (44)	2 (8)	11 (44)
Female	35 (58.3)	9 (25.7)	12 (34.3)	–	13 (37.1)
Total	60 (100)	25 (41.7)	23(38)	2 (3.3)	24 (40)

**Table 9:** Performance of microscopy and RDTs across both cohorts.

	**Symptomatic Cohort N=60**						**Asymptomatic Cohort N=200**					
	Positive	Negative	Prevalence	Sensitivity (95% CI)	Fishers P value	κ value	Positive	Negative	Prevalence	Sensitivity (95% CI)	Fishers P value	κ value
												
SD Bioline	24	36	40%	96% (0.7965–0.990)	<0.0001	0.966	34	166	64.15%	64.15% (0.4980–0.7686)	<0.0001	0.725
ACON	25	35	41.67%	100% (0.8628–1.000)	<0.0001	1.000	36	164	18%	67.92% (0.5368–0.8008)	<0.0001	0.757
Microscopy	25	35	41.67%				53	147	26.5%

**Table 10:** Test specifications.

RDT	Specification
ACON Malaria Pf/Pan test kit	HRP-2 antigen
	Aldolase antigen
SD Bioline Malaria Ag Pf test Kit	HRP-2 Antigen

**Table 11:** Contingency tables for symptomatic cohort.

	Microscopy		
SD Bioline Kit		Positive	Negative
	Positive	19	5
	Negative	6	30
	Microscopy		
ACON Malaria Pf/Pan Kit		Positive	Negative
	Positive	21	4
	Negative	4	31

**Table 12:** Contingency tables for asymptomatic cohort.

	Microscopy		
SD Bioline Kit		Positive	Negative
	Positive	34	0
	Negative	19	147
	Microscopy		
ACON Malaria Pf/Pan Kit		Positive	Negative
	Positive	33	3
	Negative	20	144

## 2 Experimental design, materials, and methods

Data was obtained between November, 2016 and May, 2017 from 260 participants, 200 asymptomatic and 60 symptomatic subjects attending the University Health Centre.

Blood samples for analysis were obtained using either of two methods; direct sampling via finger prick or venous blood collected into EDTA bottles. Tests were performed using two RDT kits (ACON Malaria P.f/ Pan Rapid Test Device and SD BIOLINE Malaria Ag P.f test kits) [1], [2], [3], [4], [5]. Thick blood smears were prepared and stained with 10% Giemsa for 15 min to determine parasitemia which was estimated from the thick film by counting the number of parasites within 200 white blood cells (leukocyte) [6], [7].

The two-tailed Fisher׳s exact test (95% Confidence Interval) was used to check for significant differences in the sensitivities of the RDTs. Inter-test agreement for positive and negative results was expressed by the percentage of overall agreement. Kappa statistic (κ) was used to determine the agreement between malaria RDTs and the reference methods. κ-values 0.6–0.8 was considered as good while k-values >0.8 were considered excellent.
